# On finite-horizon control of genetic regulatory networks with multiple hard-constraints

**DOI:** 10.1186/1752-0509-4-S2-S14

**Published:** 2010-09-13

**Authors:** Cong Yang, Ching Wai-Ki, Tsing Nam-Kiu, Leung Ho-Yin

**Affiliations:** 1 Advanced Modeling and Applied Computing, Department of Mathematics, The University of Hong Kong, Hong Kong.

## Abstract

**Background:**

Probabilistic Boolean Networks (PBNs) provide a convenient tool for studying genetic regulatory networks. There are three major approaches to develop intervention strategies: (1) resetting the state of the PBN to a desirable initial state and letting the network evolve from there, (2) changing the steady-state behavior of the genetic network by minimally altering the rule-based structure and (3) manipulating external control variables which alter the transition probabilities of the network and therefore desirably affects the dynamic evolution. Many literatures study various types of external control problems, with a common drawback of ignoring the number of times that external control(s) can be applied.

**Results:**

This paper studies the intervention problem by manipulating multiple external controls in a finite time interval in a PBN. The maximum numbers of times that each control method can be applied are given. We treat the problem as an optimization problem with multi-constraints. Here we introduce an algorithm, the "Reserving Place Algorithm'', to find all optimal intervention strategies. Given a fixed number of times that a certain control method is applied, the algorithm can provide all the sub-optimal control policies. Theoretical analysis for the upper bound of the computational cost is also given. We also develop a heuristic algorithm based on Genetic Algorithm, to find the possible optimal intervention strategy for networks of large size.

**Conclusions:**

Studying the finite-horizon control problem with multiple hard-constraints is meaningful. The problem proposed is NP-hard. The Reserving Place Algorithm can provide more than one optimal intervention strategies if there are. Moreover, the algorithm can find all the sub-optimal control strategies corresponding to the number of times that certain control method is conducted. To speed up the computational time, a heuristic algorithm based on Genetic Algorithm is proposed for genetic networks of large size.

## Background

The major goal of functional genomics is screening genes that determine specific cellular phenotypes (diseases) and modeling their activities in such a way that normal and abnormal behaviors can be differentiated. The pragmatic manifestation of the above goal is developing therapies based on the disruption or mitigation of aberrant gene function contributing to the pathology of certain disease. Mitigation would be accomplished by the use of drugs to act on the gene products. There are three things involved in engineering therapeutic tools, namely, synthesizing nonlinear dynamical networks, characterizing gene regulation and developing intervention strategies to modify dynamical behavior. In this paper, we introduce a new optimization finite-horizon control problem with multiple hard-constraints. First we review some models for studying genetic regulatory networks, Boolean networks (BNs) and Probabilistic Boolean networks (PBNs). A brief review of intervention strategies is also given. We then introduce our mathematical formulation of the problem and the Reserving Place Algorithm. The upper bound for the computational cost is also estimated. We report the results of computational experiments for different genetic regulatory networks by Reserving Place Algorithm and (or) the Genetic Algorithm. Finally, conclusions are given at the end of the paper.

### A Review on Boolean Networks (BNs) and Probabilistic Boolean Networks (PBNs)

In computational systems biology, building mathematical models and efficient numerical algorithms to study regulatory interactions among DNA, RNA, proteins and small molecules are important issues [1]. There have been many mathematical models proposed to study genetic regulatory networks such as Boolean networks (BNs) [2], regression model [3], Probabilistic Boolean Networks(PBNs) [4,5] and reviews on other mathematical models can be found in [6,7].

Among these models, BNs and its extension PBNs have received much attention as they can capture the switching behavior of the biological process [1]. Boolean networks (BNs) are introduced by Kauffman [2,8]. In a BN, each gene *i* is regarded as a vertex in the network and the gene expression state at time *t* is quantized to two levels: on and off (represented by 1 and 0). A BN consists of a set of genes {*v*_1_,*v*_2_, …, *v_n_*} and a set of Boolean functions {*f*_1_, *f*_2_, …, *f_n_*}. The Boolean function *f_i_* : {0,1}*^n^* → {0,1} represents the rules of the regulatory interactions among the genes: *v_i_*(*t* + 1) = *f_i_*(v(*t*)), *i* = 1, 2, … ,*n*. Here v(*t*) = (*v*_1_(*t*), …,*v_n_*(*t*))*^t^* is called the Gene Activity Profile (GAP). The GAP can take any possible form from the set *S* = {(*v*_1_, …, *v_n_*)*^τ^* : *v_i_* ∈ {0,1}}, thus totally there are 2*^n^* possible states. A BN is indeed a deterministic model and the only randomness comes from its initial state. Given an initial state, the BN will eventually enter into a set of state(s) called the attractor cycle and stays there forever [2]. The attractors have biological significance such as states of cell proliferation or cell apoptosis [9]. 

However, genetic regulation process exhibits uncertainty and microarray data sets used to infer the model have errors due to experimental noise in the complex measurement process. Thus it is more realistic to consider stochastic models. To extend BNs to PBNs, the main idea is as follows. To determine *v_i_* the state of gene *i* (*i* = 1, 2, …,*n*), there can be more than one Boolean functions  to be chosen. Here  is the total number of possible Boolean functions for gene *i*. The probability of choosing  as the predictor function is , where  and  One can estimate the probability  by the method Coefficient of Determination (COD) with real gene expression data sets [10]. There are at most  different possible realizations of BNs. In an independent PBN, the selection of the Boolean function for each gene is assumed to be independent, the probability of choosing the corresponding *j *th BN is given by  In fact, the transition probability matrix A can be written as the sum of the Boolean network matrices  where 0 ≤ *q_i_* ≤ 1, and  Given a PBN, assuming that the underlying Markov chain is irreducible, its long-run behavior is characterized by its steady-state probability distribution [11].

### Review of intervention strategies

Intervention strategies are applied to drive the whole genetic network into a desirable steady-state probability distribution. The intervention studies used three different approaches: (1) resetting the state of the PBN to a more desirable initial state and letting the network evolve from there, (2) changing the steady-state(long-run) behavior of the genetic network by minimally altering the rule-based structure and (3) manipulating external control variables which alter the transition probabilities of the network and therefore desirably affect the dynamic evolution. In [12], the potential effect of individual gene on the global dynamical network behavior is studied, by means of random gene perturbation and intervention. A model for random gene perturbation is given and a formula for the transition probabilities in the PBN model with perturbation is also provided. The effects of deliberately affecting a particular gene by means of intervention are also studied. A methodology for altering the steady-state probabilities of certain states or sets of states with minimal modification to the underlying rule-based structure is developed in [13]. In [14], an optimal finite-horizon control problem for gene intervention is formulated as a minimization problem with penalty costs. The penalty costs include both control cost and cost of the terminal states. The control cost is defined as the cost of applying control inputs in some particular states. Relatively higher terminal costs are assigned to those undesirable states. Other control problems such as imperfect information, context-sensitive PBN and infinite-horizon control are discussed in [15,16] separately. In [17], an algorithm is proposed to study the problem of controlling a gene network (without state feedback) such that it reaches a target state set with a prescribed maximum or minimum probability.

### Our contribution

All the above optimal control formulations did not consider the realistic hard constraints that the number of times of applying controls are bounded. In case of disease such as cancer, control inputs can be medication or radiation, etc. They are typically applied during a time period. Certain treatments such as radiation can not be applied too many times. The study in [18] fills the blank by studying an optimal finite-horizon problem with such hard-constraint. It discusses the problem with only one control variable. Observing that usually there are multiple treatment methods applied together, we study a finite-horizon external control problem with multiple hard-constraints. Our objective is to minimize the cost of control strategy over certain time period. Beside setting the upper boundaries for the number of times each control method applied, We adopt the idea that the network should fall into a desirable state set with a prescribed minimum or maximum probability from [17]. Apart from introducing the finite-horizon control problem with multiple hard-constraints, we provide an algorithm, the Reserving Place Algorithm, to generate all optimal control strategy (strategies). As the problems proposed in our paper and in [18] are both for a fixed time interval say *T*, the optimal control strategy is the sequence of control actions of length *T*. In [18], the authors start from set {0,1} which is the possible control strategies at time *t* = 1 and check if the hard-constrain is met, and recursively find those control sequences of length *T* meeting the constraint on the number of applied times. Our algorithm directly focus on control strategies of length *T*. Moreover the constraints of the numbers of times that each control method can be applied is involved in the generation of control strategies. Besides the optimal control strategy, given a fixed number of times that certain control method is applied, the algorithm can provide all the optimal control sequences. We remark that our proposed formulation can be applied to both perturbed and context-sensitive PBNs, though we only discuss examples of instantaneously random PBNs.

## Methods

We first give some necessary notations to introduce the mathematical formulation of our optimization control problem. We then describe an algorithm, the Reserving Place Algorithm, for obtaining all the optimal solutions. The upper bound of computational cost is also estimated. Based on this, the drawbacks of the Reserving Place Algorithm is stated and we apply the Genetic Algorithm to networks of large size. Here we study an optimization control problem with multiple hard-constraints. Our goal is to find an optimal strategy for manipulating external control variables to desirably affect the dynamic evolution of a random PBN over a finite time horizon with minimum corresponding cost. Without loss of generality, here we only consider two control methods. At each time point *t* (*t* = 1, 2, …, *T*), one of the following three control options will be conducted: Control 0 (i.e. no control), Control 1 and Control 2, represented by *u*_0_, *u*_1_ and *u*_2_. Their corresponding transition probability matrices *P*_0_, *P*_1_, *P*_2_ are given. The optimal control problem can be stated as follows. Given an initial probability distribution x_0_ = (*v*_1_(0), …,*v_n_*(0))^*t*^ and a set of target states *S*′ ⊆ *S*, our goal is to find a sequence of actions *σ* over a finite time horizon *T* that leads the system reaching a target state with a minimum probability (i.e  where [x*_T_*]*_i_* = *v_i_*(*T*) is the state of gene *i* at time *T*) while minimizing the sum of the costs of the control actions applied at each time point  Thus we obtain the following optimization control problem:

 (1)

Here *s_i_* is the number of times that Control *i* is conducted, and *K_i_* is the maximum number of times that Control *i* can be applied, *i* = 1, 2. We use *i_j_* ∈ {0,1, 2} to represent that Control *i_j_* is applied to the network at time *j*. Then the control string *i*_1_*i*_2_ …, *i_k_* represents the control actions conducted from time 1 to time *k*. We define set *U* = {*σ* = *i*_1_*i*_2_ … *i_T_* : *i_j_* ∈ {0,1, 2}, and 0 ≤ *s_i_* ≤ *K_i_*, *i* =1, 2} as the set containing all the possible control strategies satisfying the multiple hard-constraints. Given the initial probability distribution vector x_0_, the state probability distribution vector  represents the state vdistribution vector at time *T* obtained by control strategy *σ* = *i*_1_*i*_2_ … *i_T_*. The feasible solution set *V* is a subset of set *U*, where

Optimal solution(s) exists(exist) if *V* is not empty.

### Algorithms

#### *Reserving place algorithm*

Our proposed problem is NP-hard. Here we develop an algorithm for computing all the optimal solutions. In order to find the feasible solution set for the optimal control problem with hard-constraint, [18] applied a recursive method as follows. They first start with the set {0, 1}, which contains all the possible control strategies at time *t* = 1. Then one can obtain the set {00, 01, 10, 11} for time *t* = 2. Recursively one can get the feasible solution set while checking whether the control strategies satisfy the hard-constraint. The problem proposed in this paper involves more than one control methods under multiple hard-constraints. The recursive method in [18] can be applied. Here we introduce a more efficient algorithm, the Reserving Place Algorithm to find the feasible solution set  Our algorithm focuses on control strategies of length *T* at the right start. For the generation of set *U*, the numbers of times that each control method can be applied are also involved into consideration. Then one only need to check whether the state probability distribution obtained by any control string in the set *U* satisfies the constraint  Thus the key point is to generate the set *U*, the set of all possible control strategies satisfying the hard-constraints.

We first assume that the number of times that Control 2 is applied is fixed as *k*, 0 ≤ *k* ≤ *K*_2_. We reserve k places in the control string of length *T* for Control 2, then there are totally  cases. Now we only need to find all the control strings of length *T* − *k* where Control 0 (i.e. no control) and Control 1 can be applied and the maximum number of times that Control 1 can be applied is *K*_1_. We note that among all the possible control strings, binary string  is the biggest one. Thus by translating decimal digits from 0 to 2*^T−k^* − 1 to binary digits and checking the number of times that Control 1 is applied, one can generate all the control strings of length *T* − *k* satisfying the hard-constraint for Control 1. Finally we can obtain the set *U* by increasing *k* from 0 to *K*_2_.

#### *Computational cost*

Here we provide an upper bound of the computational cost for our Reserving Place Algorithm.

**Theorem 1***The computation cost of the Reserving Place Algorithm is bounded above by MT*2^2^*^n^ where*

**Proof:** The main computational cost of the algorithm comes from the matrix-vector multiplication. For each control strategy, the number of matrix-vector multiplication is *T*, where *T* is the length of time interval. If we search an optimal solution in the set *W* = {*σ* = *i*_1_*i*_2_ … *i_T_* : *i_j_* ∈ {0,1, 2}} the computational cost is *T*3*^τ^*2^2^*^n^*, where *n* is the number of genes in the network. Since we only need to consider the strategies in the set , the computational cost is *T*2^2^*^n^n*(*V*), where *n*(*V*) is the number of control strategies in the set *V*. We note that *V* ⊆ *U*, the computational cost is bounded above by *T*2^*2n*^*n*(*U*). By the Reserving Place algorithm, we have

	(2)

#### *Genetic algorithm*

It has been shown that finding a control strategy for a BN to a global state is actually NP-hard [19]. By Theorem 1, we know that the computational cost of the Reserving Place Algorithm depends a lot on the length of the time interval *T* and the number of genes *n*. Note that the number of possible states in the network increases exponentially with respect to the number of genes *n*, thus the computational cost for solving the optimal control problem can be enormous even for moderate *n*. For any of the above two cases, we apply the Genetic Algorithm (GA) for the proposed multiple hard-constraint problem. GA is inspired by evolutionary biology such as inheritance, mutation, selection, and crossover. It is a search technique used in computing to find exact or approximate solutions to optimization and search problems. In the first step, we generate a random population of size *N* = 100, consisting policy vectors of length *T* = 100. The cost of each policy vector is evaluated which is subsequently turned into the probability that it would be picked for the next generation. Second, we pick 2 policies from the current generation with replacement according to their corresponding probabilities. Then, with cross over rate p_c_ = 0.7, crossover of the two policies occurs at a random position. After that, each position of the policies mutates with mutation rate p_m_ = 0.01. After the above operations, the two resulting vectors are placed in the new population. Two policies are picked at a time from the current population and then the crossover and mutation operations are performed whenever necessary until there are *N* or *N* +1 policies in the new generation. We then calculated the cost and the probability of each policy vector. If *N* is odd, we randomly remove one policy vector from the new generation. The cost and probability of each policy vector are then calculated. The process returns to the second step unless the stopping criteria is met. Since the GA starts by randomly generating an initial population, it cannot guarantee to obtain an optimal solution. Thus to obtain a reasonably good solution, in numerical experiments, we apply GA many times, and obtain an optimal solution from all the results obtained.

## Results

This section is organized into three parts. First, we provide a 2-gene hypothetical genetic network. Both the Reserving Place Algorithm and Genetic Algorithm are applied. The contrast in computational time is also given. Then both algorithms are applied to a 3-gene hypothetical genetic network. Finally, the comparison of the two algorithm is conducted.

### 2-gene network

We start with a 2-gene hypothetical genetic network. The network consists of two genes denoted by *A* and *B*. The states of gene *A* and gene *B* are given in Table 1. There are three external control methods: (i) Control 0: no control, (ii) Control 1: medication, and (iii) Control 2: radiation. Their corresponding transition probability matrices are given as follows.

	(3)

**Table 1:** States of Genes in the 2-gene network

States	1	2	3	4
*A*	Off	Off	On	On
*B*	Off	On	Off	On

Our objective is to find a control strategy that ensures after time length *T* the total probability of gene *A* being expressed is at least 0.8 (i.e., [x]_3_ + [x]_4_ ≥ 0.8) with the minimum cost, given an initial state distribution of x_0_ = (0.1, 0.4, 0.3, 0.2)*^t^*. The maximum numbers of times that Control 1 and Control 2 can be conducted are *K*_1_ =5 and *K*_2_ = 2 respectively. The cost for conducting Control 1 is 2.5, the cost for Control 2 is 4, and no cost for Control 0. We first apply the Reserving Place Algorithm to the 2-gene network when *T* = 10. Table 2 lists the obtained sub-optimal strategies with minimum cost for each fixed *k* from 0 to *K*_2_ = 2, where *k* is the number of times that Control 2 is conducted. From Table 2, there is only one optimal control strategy: conduct Control 2 at time point *t* = 7 and Control 1 at time point *t* = 8, 9,10, with total cost 11.5 and corresponding state probability distribution vector x*_T_* = (0.0275, 0.1682,0.2679,0.5364)*^t^*. For various values of time interval length *T*, Table 3 provides the corresponding optimal strategies and their costs by Genetic Algorithm. The Genetic Algorithm cannot always find the optimal solution due to the random initial guess. Here we repeatedly apply the GA 50 times, and the control strategy is chosen from those results. The GA algorithm can also obtain (only one) the optimal solution obtained by the Reserving Place Algorithm. To contrast the computational cost, Table 4 gives the computing time for the Reserving Place Algorithm and the average computing time for the Genetic Algorithm for the 2-gene network under various values of *T*.

**Table 2:** Sub-optimal solutions for 2-gene example (*T* = 10) by Reserving Place Algorithm

***k***	Control Strategy *σ = = i_1_i_2_ …, i_T_*										Cost	Computing Time
	0	0	0	1	0	0	1	1	1	1		
*k* = 0	0	0	0	0	1	0	1	1	1	1	12.5	0.156
	0	0	0	0	0	1	1	1	1	1		

*k* = 1	0	0	0	0	0	0	2	1	1	1	11.5	0.718

	0	0	0	0	0	2	2	1	1	1		
	0	0	0	0	2	0	2	1	1	1		
*k* = 2	0	0	0	2	0	0	2	1	1	1	15.5	9.375
	0	0	2	0	0	0	2	1	1	1		
	0	2	0	0	0	0	2	1	1	1		
	2	0	0	0	0	0	2	1	1	1		

**Table 3:** Optimal solutions for 2-gene example under various T by Genetic Algorithm

	Control Strategy *σ = i_1_i_2_ …, i_T_*															Cost
*T* = 10						0	0	0	0	0	0	2	1	1	1	11.5
*T* = 11					0	0	0	0	0	0	0	2	1	1	1	11.5
*T* = 12				0	0	0	0	0	0	0	0	2	1	1	1	11.5
*T* = 13			0	0	0	0	0	0	0	0	0	2	1	1	1	11.5
*T* = 14		0	0	0	0	0	0	0	0	0	0	2	1	1	1	11.5
*T* = 15	0	0	0	0	0	0	0	0	0	0	0	2	1	1	1	11.5

**Table 4:** Comparison of computing time of the two algorithms

	Reserving Place Algorithm(sec)	Genetic Algorithm(sec)
*T* =10	10.3	29.6
*T* =11	68.8	29.9
*T* =12	315.2	30.1
*T* =13	1177.0	28.9
*T* =14	4017.9	30.0
*T* =15	10796.0	29.5

### 3-gene network

Here we consider a hypothetical network consisting of 3 genes A, B, C. The states of genes *A*, *B* and *C* are given in Table 5. There are three external control methods: (i) Control 0: no control, (ii) Control 1: medication, and (iii) Control 2: radiation. Their corresponding transition probability matrices are given as follows.

 (4)

**Table 5:** States of Genes in the 3-gene network

States	1	2	3	4	5	6	7	8
*A*	Off	Off	Off	Off	On	On	On	On
*B*	Off	Off	On	On	Off	Off	On	On
*C*	Off	On	Off	On	Off	On	Off	On

Our objective is to find a control strategy that ensures the total probability of gene *A* unexpressed and gene *B* expressed is at least 0.8 (i.e., [x]_3_ + [x]_4_ ≥ 0.8) with the minimum cost, given an initial state distribution of x_0_ = (0.1, 0.2, 0.1, 0.1, 0.25, 0.15, 0.1, 0)*^t^*. The maximum numbers of times that Control 1 and Control 2 can be conducted are *K*_1_ = 5 and *K*_2_ = 2 respectively. The cost for conducting Control 1 is 2.5, the cost for Control 2 is 4, and no cost for Control 0.

Table 6 gives the obtained sub-optimal strategies with minimum cost for each fixed *k* from 0 to *K*_2_ = 2, where *k* is the number of times that Control 2 is conducted by the Reserving Place Algorithm. The length of time interval is *T* = 10. As listed in Table 6, there are two optimal control strategies both with minimum cost 6.5. The computing time for the Reserving Place Algorithm is 6.94 sec. For the case of *T* = 15, the computing time is 8450.4 sec. In Table 7 we present the best control strategies obtained by Genetic Algorithm and also its corresponding average computing time for time intervals *T* = 10,15, 20.

**Table 6:** Sub-optimal solutions for 3-gene example (*T* = 10) by Reserving Place Algorithm

***k***	Control Strategy *σ = i_1_i_2_ …, i_T_*										Cost	Computing Time
*k* = 0					-						-	0.08

*k* = 1	0	0	0	0	0	0	0	1	0	2	6.5	0.644
	0	0	0	0	0	0	0	0	2	1		

*k* = 2	0	0	0	0	0	0	0	2	0	2	8	6.22

**Table 7:** Optimal solutions for 3-gene example under various *T* by Genetic Algorithm

	Control Strategy *σ = i_1_i_2_ …i_T_*																				Cost	Average Computing Time(sec)
*T* =10										0	0		0	0	0	0	0	0	2	1	6.5	32.25

*T* =15						0	0	0	0	0	0	0	0	0	0	0	0	0	0	2	4	30.23

*T* = 20	0	0	0	0	0	0	0	0	0	0	0	0	0	0	0	0	0	0	0	2	4	27.7

### A comparison of the two algorithms

Based on the numerical experiments, we draw the following remarks for the comparison of the Reserving Place Algorithm and the Genetic Algorithm. The Reserving Place Algorithm obtains all the optimal control strategies, meanwhile the Genetic Algorithm provides one possible optimal solution. Moreover, the Reserving Place Algorithm can give all the sub-optimal control strategies for a fixed number of times that certain control method is applied. This is useful in practice as the results can provide more applicable control strategies to be chosen and more information about the effects of combining various control methods. In the aspect of computing time, the computing time of the Reserving Place Algorithm is closely corresponding to the length of time interval *T* as shown in Table 4. On the other hand, the average computing time for the Genetic Algorithm is not much influenced by the increase of *T*. By Theorem 1, the computational time of the Reserving Place Algorithm increases exponentially with respect to the number of genes *n*. For the Genetic Algorithm, the computing time depends on *n*, but not as greatly as the computational cost of the Reserving Place Algorithm. All numerical experiments were performed via MATLAB 7.60 in Window XP using an Intel 3.20 GHz processor.

## Conclusions

In this paper, we introduced a new optimal finite-horizon control problem with multiple hard-constraints. We proposed an algorithm, the Reserving Place Algorithm, to generate all optimal solutions. The upper bound for the computational cost was also estimated. We remark that our formulation can be applied to both perturbed and context-sensitive PBNs.

## Competing interests

The authors declare that they have no competing interests.

## Authors' contributions

Wai-Ki proposed the optimization problem. Yang designed and analyzed the Reserving Place Algorithm, performed the numerical experiments. Yang, Wai-Ki and Nam-Kiu wrote the manuscript. Wai-Ki and Ho-Yin contributed to the numerical experiment analysis and modification of the manuscript. All authors have read and approved the final version of the manuscript.
